# Kinetics of Isomerization and Inversion of Aspartate 58 of αA-Crystallin Peptide Mimics under Physiological Conditions

**DOI:** 10.1371/journal.pone.0058515

**Published:** 2013-03-07

**Authors:** Kenzo Aki, Norihiko Fujii, Noriko Fujii

**Affiliations:** Research Reactor Institute, Kyoto University, Kumatori, Osaka, Japan; Russian Academy of Sciences, Institute for Biological Instrumentation, Russian Federation

## Abstract

Although proteins consist exclusively of L-amino acids, we have reported that aspartyl (Asp) 58 and Asp 151 residues of αA-crystallin of eye lenses from elderly cataract donors are highly inverted and isomerized to D-β, D-α and L-β-Asp residues through succinimide intermediates. Of these Asp isomers, large amounts of D-β- and L-β-isomers are present but the amount of D-α-isomer is not significant. The difference in abundance of the Asp isomers in the protein may be due to the rate constants for the formation of the isomers. However, the kinetics have not been well defined. Therefore, in this study, we synthesized a peptide corresponding to human αA-crystallin residues 55 to 65 (T^55^VLD^58^SGISEVR^65^) and its isomers in which L-α-Asp at position 58 was replaced with L-β-, D-β- and D-α-Asp and determined the rate of isomerization and inversion of Asp residues under physiological conditions (37°C, pH7.4). The rate constant for dehydration from L-α-Asp peptide to L-succinimidyl peptide was 3 times higher than the rate constant for dehydration from L-β-Asp peptide to L-succinimidyl peptide. The rate constant for hydrolysis from L-succinimidyl peptide to L-β-Asp peptide was about 5 times higher than the rate constant for hydrolysis from L-succinimidyl peptide to L-α-Asp peptide. The rate constant for dehydration from L-α-Asp peptide to L-succinimidyl peptide was 2 times higher than the rate constant for dehydration from D-α-Asp peptide to D-succinimidyl peptide. The rate constants for hydrolysis from L-succinimidyl peptide to L-β-Asp peptide and for hydrolysis from D-succinimidyl peptide to D-β-Asp peptide were almost equal. Using these rate constants, we calculated the change in the abundance ratios of the 4 Asp isomers during a human lifespan. This result is consistent with the fact that isomerized Asp residues accumulate in proteins during the ageing process.

## Introduction

Proteins consist exclusively of L-amino acids in living tissues. However, D-aspartyl (Asp) residues have been found in various proteins of lens[1]–[4], retina [5], conjunctivae [6], cornea of eyes [7], tooth [8], aorta [9], brain [10], [11], bone [12], [13], ligament [14] and skin [15], [16] in elderly donors. The presence of D-Asp in aged tissues of living organisms is thought to be a result of the racemization of aspartyl residues in polypeptide where the proteins in such tissues are metabolically inert. Of all the naturally occurring amino acids, aspartic acid (Asp) is the most susceptible to racemization. The aspartyl residues are racemized non-uniformly presumably because of structural considerations, which make specific residues more susceptible to reaction than others. However, there are few papers describing the determination of the specific sites at which racemization of Asp residues in proteins occurs. Those studies that have been published identified specific sites of D-Asp in αA-crystallin [2], αB- crystallin [3] andβB2-crystallins [4] from lens, the β-amyloid protein in brain [11], histone of canine brain [17] and type I collagen tellopeptide in urine [13].

**Figure 1:**
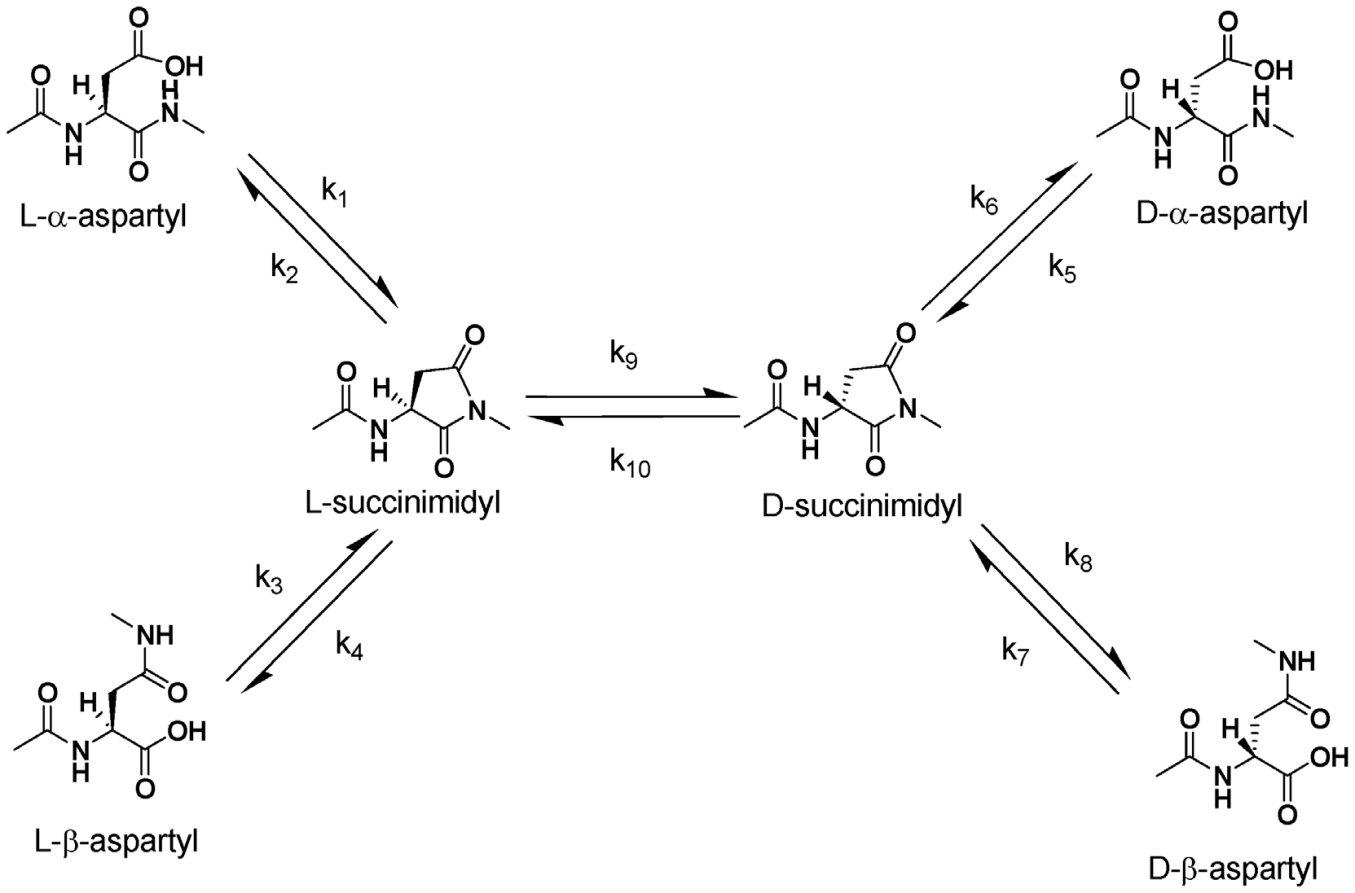
Pathways for spontaneous isomerization and racemization of Asp residues in a peptide. k1–k10 represent the rate constants for each reaction.

D-Asp formation is also accompanied by isomerization from the natural α-Asp to the biologically uncommon β-Asp form [18]. Therefore, the inversion of Asp occurs *via* a succinimide intermediate as follows: (i) When the carbonyl group of the side chain of the L-α-Asp residue is attacked by the nitrogen of the amino acid residue following the Asp residue, L-succinimide is formed by intramolecular cyclization; (ii) L-succinimide may be converted to D-succinimide through an intermediate that has the prochiral α-carbon in the plane of the ring; (iii) The D- and L-succinimide are hydrolyzed at either side of their two carbonyl groups, yielding both α- and β-Asp residues, respectively. Thus, 4 isomers, L-α-Asp, L-β-Asp, D-α-Asp and D-β-Asp, are simultaneously formed in the protein (Figure 1). The racemization of the amino acid residue is caused by abstraction of the proton attached to the α-carbon. Generally, the proton attached to the α-carbon of amino acids is not released under physiological conditions. However, the proton attached to the α-carbon of Asp residueis released more easily than that of the other amino acids because the Asp residue converts to succinimide from which the proton is readily released. Therefore, the susceptibility of the racemization of Asp is due to that of the formation of the succinimidyl intermediate.

In our previous studies, we found that specific Asp residues in αA-crystallin (Asp 58 and Asp 151) [2], αB-crystallin (Asp 36 and Asp 62) [3] and βB2-crsytallin (Asp 4) [4] in the human lens were highly inverted from the L-isomer to the D-isomer and the peptide bond between Asp and the next amino acid was isomerized from the normal α-linkage to a β-linkage. The appearance of these isomers may cause major changes to the protein structure, since different side chain orientations can induce an abnormal peptide backbone. Therefore, the presence of the isomers may be one of triggers of abnormal aggregation and can induce the partial unfolding of protein leading to a disease state. In fact, the previous study clearly showed that α-crystallin containing large amounts of D-β-Asp undergoes abnormal aggregation to form massive and heterogeneous aggregates, leading to loss of its chaperone activity [19]. Similarly, we observed the accumulation of D-β-Asp containing proteins in sun-damaged skin from the faces of elderly people [15]. The abnormal protein was localized to the elastic fiber-like structures of the dermis in elderly donors with actinic elastosis [20]. The D-β-Asp containing proteins are not metabolized by catabolic enzymes because the enzymes are composed of L-amino acids which do not recognize the D-β-Asp containing proteins. In addition, no enzyme is known which recognizes and repairs the D-β-Asp residues. Therefore the D-β-Asp containing proteins accumulate in the various tissues. For these reasons the abnormal Asp residues are present widely and arise due to racemization of amino acids in various proteins during the lifespan of the individual. Therefore, it is necessary to predict the relative amounts of the 4 Asp isomers during aging. Here we report the kinetics of isomerization and racemization of Asp-58 in a model synthetic peptide corresponding to a peptide encompassing residues 55 to 65 of αA-crystallin.

## Results

### The Synthesis of T6-Lα, T6-Lβ, T6-Dα and T6-Dβ and their Separation by RP-HPLC Chromatography

**Figure 2:**
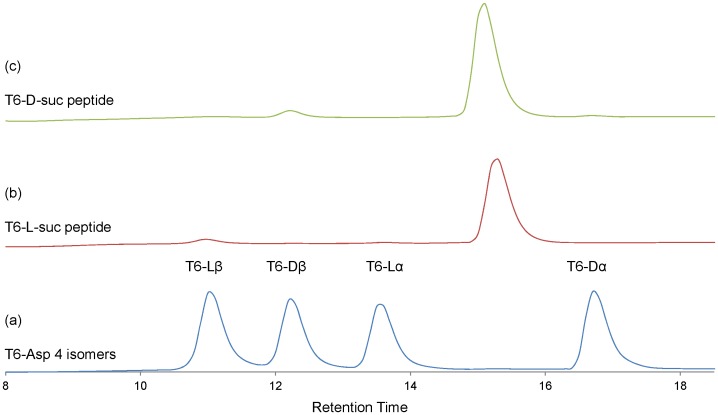
The overlay of elution profiles of the purified T6-D-suc, T6-L-suc and the mixture of synthesized T6 Lα, T6 Lβ, T6 Dα, T6 Dβ peptides. Column: Shiseido UG 80 3.0×250 mm. Gradient: 17–26.5% acetonitrile/0.1% TFA over 25 min. Flow rate: 0.5 mL/min. Detection: 215 nm. a: elution profile of mixture of synthesized T6 Lα, T6 Lβ, T6 Dα and T6 Dβ. b: elution profile of purified T6-L-suc. c: elution profile of purified T6-D-suc.

We synthesized the T6 peptide corresponding to human αA-crystallin from residues 55 to 65 (T^55^VLD^58^SGISEVR^65^) and its isomers in which L-α-Asp at position 58 was replaced with L-β-, D-β- and D-α-Asp by Fmoc (9-fluorenylmethoxycarbonyl) solid-phase chemistry using an automated solid-phase peptide synthesizer. These peptides were designated T6-Lα, T6-Lβ, T6-Dα and T6-Dβ (Table 1). Figure 2a shows the RP-HPLC chromatograms of T6-Lα, T6-Lβ, T6-Dα and T6-Dβ peptides. These peptides are clearly separated into 4 peaks in the RP-HPLC chromatogram due to the presence of the different Asp isomers. Therefore, the relative amounts of the T6-Lα, T6-Lβ, T6-Dα and T6-Dβ peptides can be estimated from the peak are as shown in the chromatogram.

**Table 1:** T6 and its related peptides synthesized in this study.

Sequence	peptide	M^+^H
Thr^55−^Val-Leu- L-α-Asp^58^-Ser-Gly-Ile-Ser-Glu-Val-Arg^65^	T6-Lα	1175.3
Thr^55−^Val-Leu- L-β-Asp^58^-Ser-Gly-Ile-Ser-Glu-Val-Arg^65^	T6-Lβ	1175.3
Thr^55−^Val-Leu- D-α-Asp^58^-Ser-Gly-Ile-Ser-Glu-Val-Arg^65^	T6-Dα	1175.3
Thr^55−^Val-Leu- D-β-Asp^58^-Ser-Gly-Ile-Ser-Glu-Val-Arg^65^	T6-Dβ	1175.3
Thr^55−^Val-Leu- L-suc^58^-Ser-Gly-Ile-Ser-Glu-Val-Arg^65^	T6-L-suc	1157.3
Thr^55−^Val-Leu-D-suc^58^-Ser-Gly-Ile-Ser-Glu-Val-Arg^65^	T6-D-suc	1157.3

### The Preparation and Purification of T6-L-suc and T6-D-suc Peptides

Both the L-succinimidyl and D-succinimidyl (T6-L-suc and T6-D-suc) peptides were obtained by heating T6-Lα and T6-Dα peptides at 90°C for 6 h in water The purified T6-suc-peptide eluted between T6-Lα and T6-Dα as shown in Figure 2. However, the formation of T6-L-suc and T6-D-suc was not observed in phosphate buffer under the same heating conditions (data not shown). The T6-L-suc-peptide and T6-D-suc-peptide were identified by mass spectroscopy. The masses observed for the protonated precursor ions of the peptides were 1157.3, which is consistent with the theoretical mass (Table 1).

### The Determination of the Rate Constants (k2, k4, k6 and k8) from the Hydrolysis of T6-L-suc and T6-D-suc Peptides

**Figure 3:**
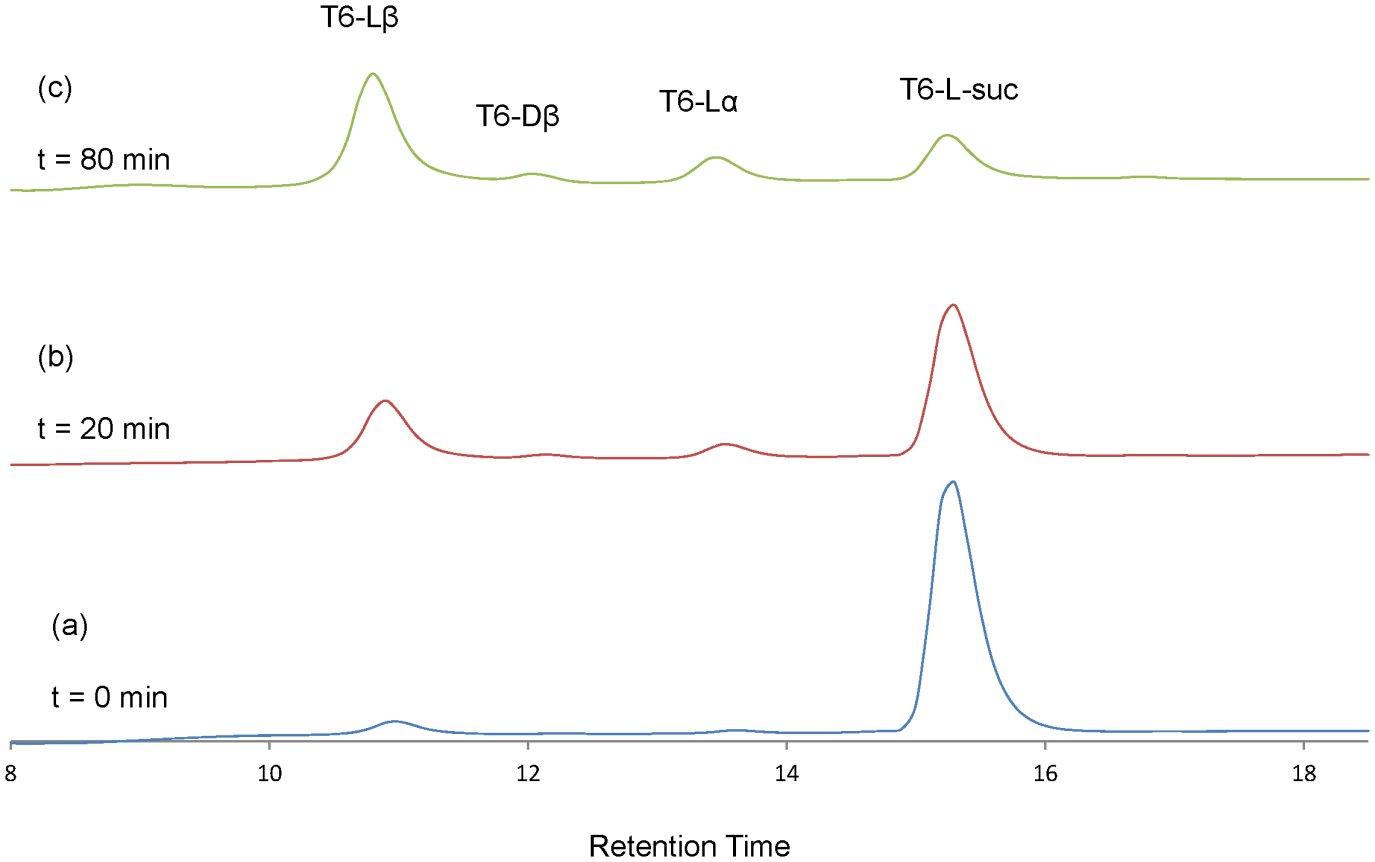
The overlay of elution profiles of T6-L-suc peptide at 0–80 min after incubation. Column: Shiseido UG 80 3.0×250 mm. Gradient: 17–26.5% acetonitrile/0.1% TFA over 25 min. Flow rate: 0.5 mL/min. Detection: 215 nm. a: elution profile of T6-L-suc peptide at incubation time = 0 min. b: elution profile of T6-L-suc peptide which was incubated at 37°C for 20 min. c: elution profile of T6-L-suc peptide which was incubated at 37°C for 80 min.

**Figure 4:**
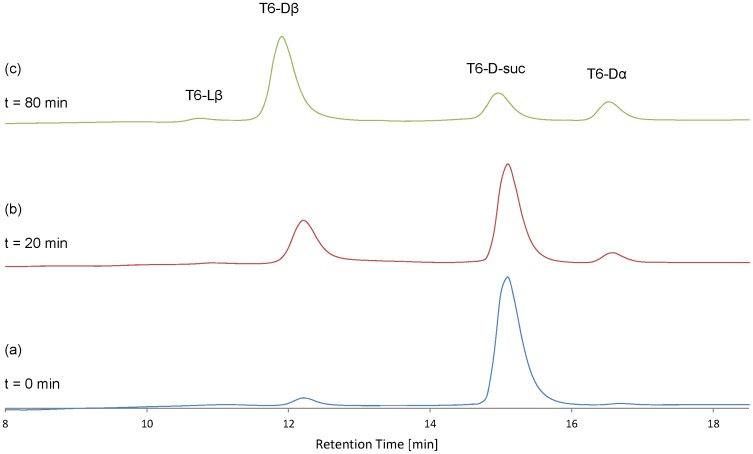
The overlay of elution profiles of incubated T6-D-suc peptide after incubation for 0–80 min. Column: Shiseido UG 80 3.0×250 mm. Gradient: 17–26.5% acetonitrile/0.1% TFA over 25 min. Flow rate: 0.5 mL/min. Detection: 215 nm. a: elution profile of T6-D-suc peptide at incubation time = 0 min. b: elution profile of T6-D-suc peptide which was incubated at 37°C for 20 min. c: elution profile of T6-D-suc peptide which was incubated at 37°C for 80 min.

T6-L-suc and T6-D-suc peptides were incubated at 37°C under physiological conditions. Figure 3a shows a RP-HPLC elution profile of T6-L-suc peptide incubated for 0 min. The T6-L-suc peptide was slightly hydrolyzed in the absence of heating due to the instability of the succinimide. Figure 3b shows the elution profile of T6-L-suc peptide incubated at 37°C for 20 min. The T6-L-suc peptide was hydrolyzed rapidly and T6- Lβ increased markedly. Figure 3c shows the elution profile of T6-L-suc peptide incubated at 37°C for 80 min. At this time point, T6-Lβ was the main component. Similarly, the T6-D-suc peptide was also hydrolyzed rapidly with T6- Dβ increasing noticeably (Figure 4).

**Figure 5:**
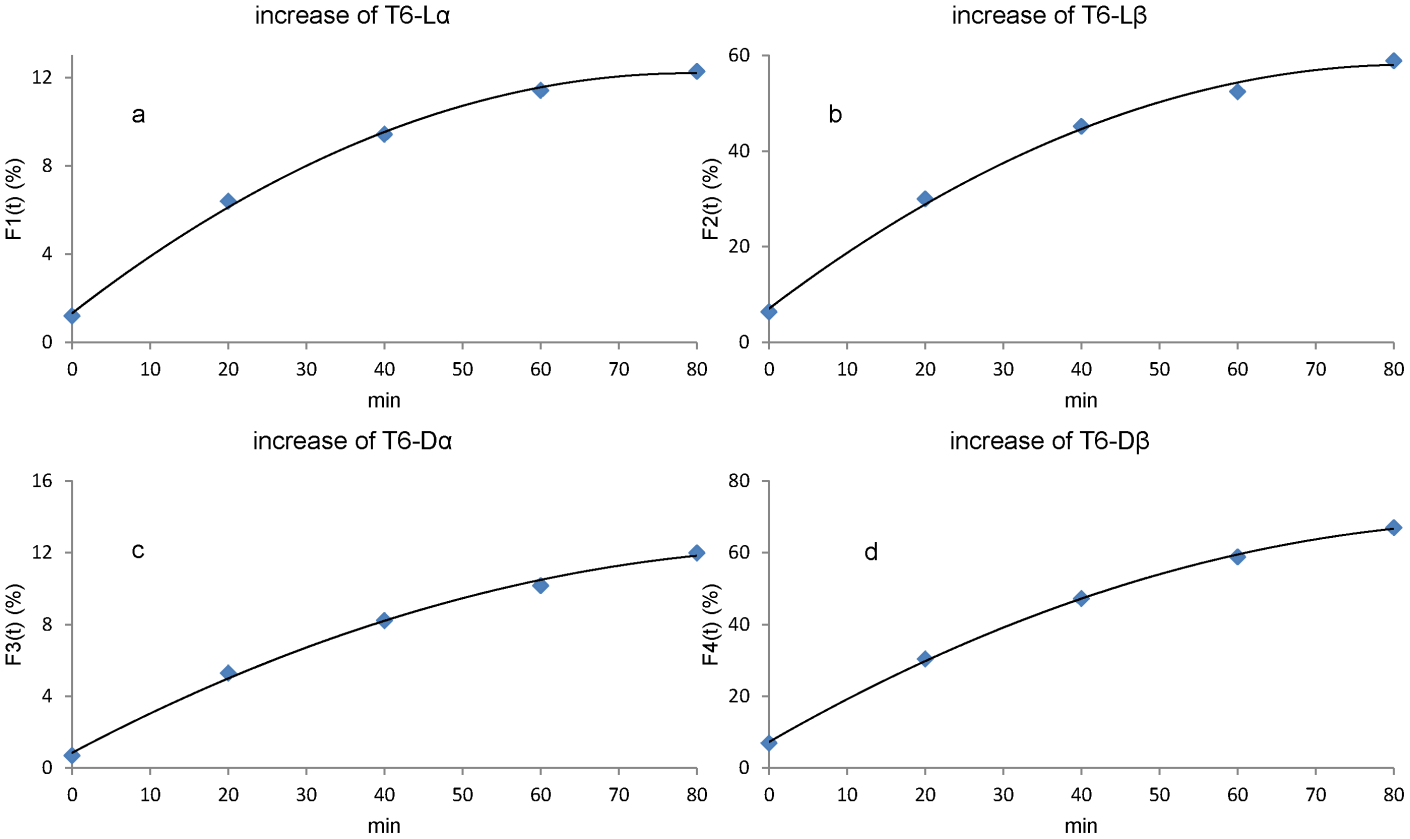
Increase of α- and β- Asp with hydrolysis of succinimide. Figure 5a: increase of L-α-Asp with hydrolysis of T6-L-suc peptide at 37°C. Figure 5b: increase of L-β-Asp with hydrolysis of T6-L-suc at 37°C. Figure 5c: increase of D-α-Asp with hydrolysis of T6-D-suc peptide at 37°C. Figure 5d: increase of D-β-Asp with hydrolysis of T6-D-suc peptide at 37°C. We calculated the rate constant for hydrolysis of succinimide at 37°C (k2, k4, k6 and k8) by using fitted curves in this figure.

Figures 5a and 5b show the formation of Lα, Lβ by the hydrolysis of the L-succinimide from the T6-L-suc peptide at 37°C. Figures 5c and 5d show the formation of Dα, Dβ by the hydrolysis of the D-succinimide from the T6-D-suc peptide at 37°C. We calculated the rate constants, k2, k4, k6 and k8 as defined in Figure 1 for hydrolysis of succinimide at 37°C by using a fitted curve as shown in Figures 5a–d. As an example, the calculation of k2 using (F1(t)) in Figure 5a is as follows;
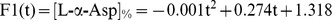
(1 - 1)where units of t are min.

Therefore, the derivative of the fitted curve is
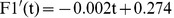
(1-2)


On the other hand, F1′(t) is expressed as F1′(t) = d[L-α-Asp]_%_/dt, therefore, d[L-α-Asp]_%_/dt can be expressed as follows:
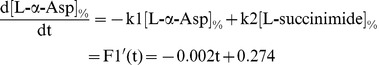
(1 - 3)where the units of k1 and k2 are min^−1^, because the units of t are min.

At t = 0 min, as compared with k2[L-succinimide]_%_, −k1[L-α-Asp]_%_which was almost equal to 0. The experimental value of [L-succinimide]_%_ was 92.415% at t = 0 min.

Therefore
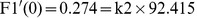
(1 - 4)Therefore, k2 was estimated to be 0.00296 min^−1^ (4.27 day^−1^).

k4, k6 and k8 were determined by the same method using the fitted curves in Figures 5b, 5c and 5d, respectively. Hydrolysis to L-β-Asp from L-succinimide is about 4.6 times faster than hydrolysis to L-α-Asp from L-succinimide. Hydrolysis to D-β-Asp from D-succinimide is also 5.5 times higher than hydrolysis to D-α-Asp from D-succinimide. The difference in hydrolysis rate of L-succinimide from D-succinimide is not so significant. The rate constants, k2, k4, k6 and k8 are summarized in Table 2.

**Table 2:** Summary of rate constants (day^−1^) under physiological conditions (37°C, pH 7.4).

k1	k2	k3	k4	k5	k6	k7	k8	k9	k10
2.22×10^−3^	4.27	0.729×10^−3^	19.3	1.20×10^−3^	3.59	0.472×10^−3^	19.6	0.529	0.543

### The Determination of the Rate Constants (k9,k10) for the Racemization of Succinimide

**Figure 6:**
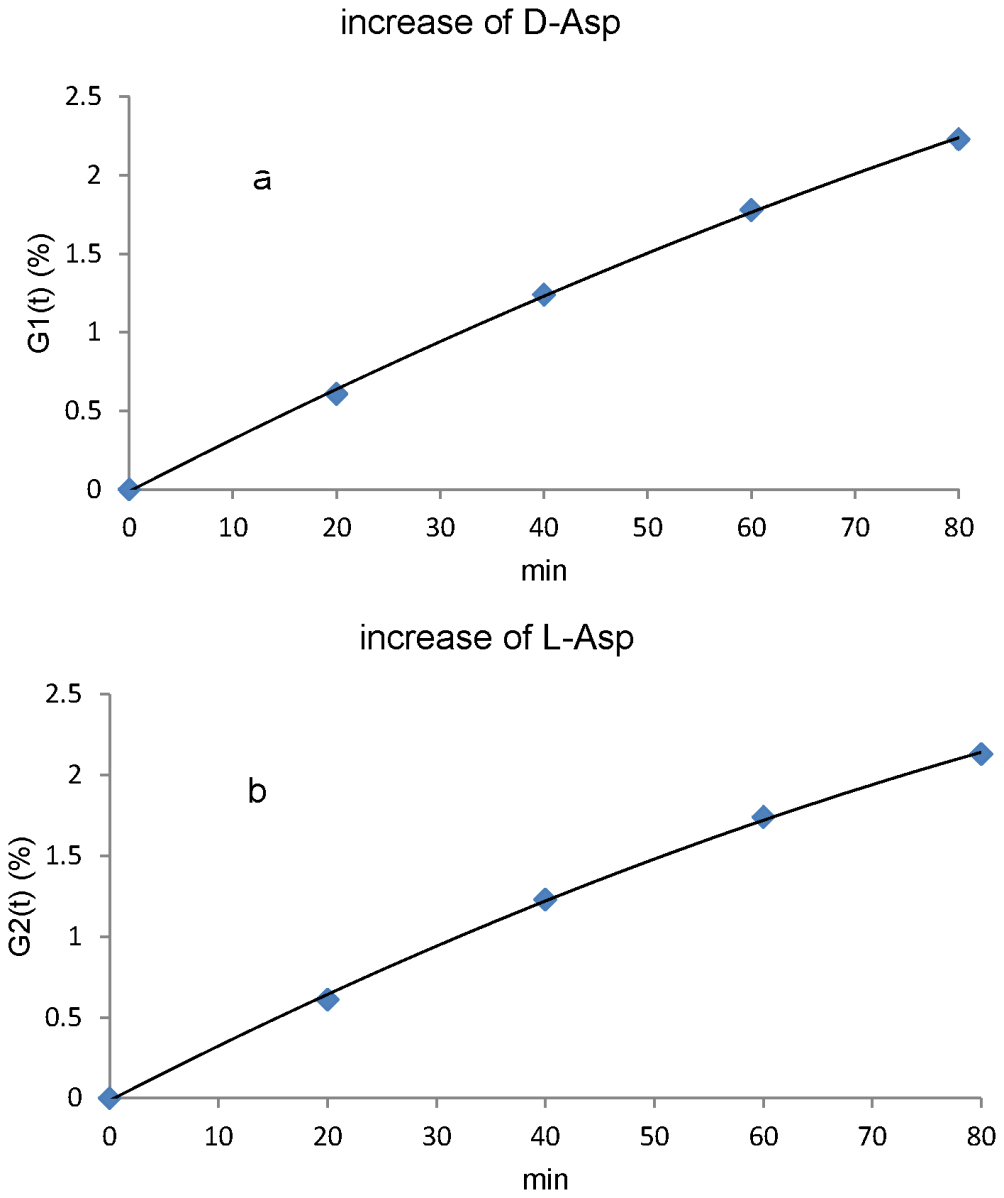
Racemization of succinimide. Figure 6a: increase of D-Asp with racemization of L-suc peptide at 37°C. Figure 6b: increase of L-Asp with racemization of D-suc peptide at 37°C. We calculated the rate constant for racemization of succinimide at 37°C (k9 and k10) by using fitted curves in this figure.

Figure 6a shows the increase of T6-D-Asp peptide (D-α-Asp+ D-β-Asp) caused by heating of T6-L-suc peptide at 37°C. Figure 6b shows the increase of T6-L-Asp-peptide (L-α-Asp+ L-β-Asp) caused by heating of T6-L-suc peptide at 37°Citself produced from the T6-D-suc peptide. We determined the racemization rate constants, k9, k10 from the fitted curves in Figure 6a and 6b. As an example, the calculation of k9 using the fitted curve (G1(t)) in Figure 6a is described below:
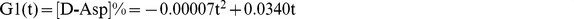
(2 - 1)where the units of t are min.

Therefore, the derivative of the fitted curve is
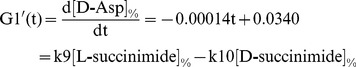
(2 - 2)where the units of k9 and k10 are min^−1^, because the units of t are min.

At t = 0 min, [D-succinimide]_%_ was almost equal to 0%. The experimental value of [L-succinimide]_%_ was 92.415% at t = 0 min. Therefore
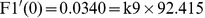
(2 - 3)Therefore, k9 is 0.000368 min^−1^ (0.529 day^−1^). k10 was determined by the same method (Table 2). The rate constants for conversion of L-succinimide to D-succinimide and the rate constant for conversion of D-succinimide to L-succinimide were almost equal.

### The Determination of the Rate Constants (k1, k3, k5, k7) for Succinimide Formation in Peptides

**Figure 7:**
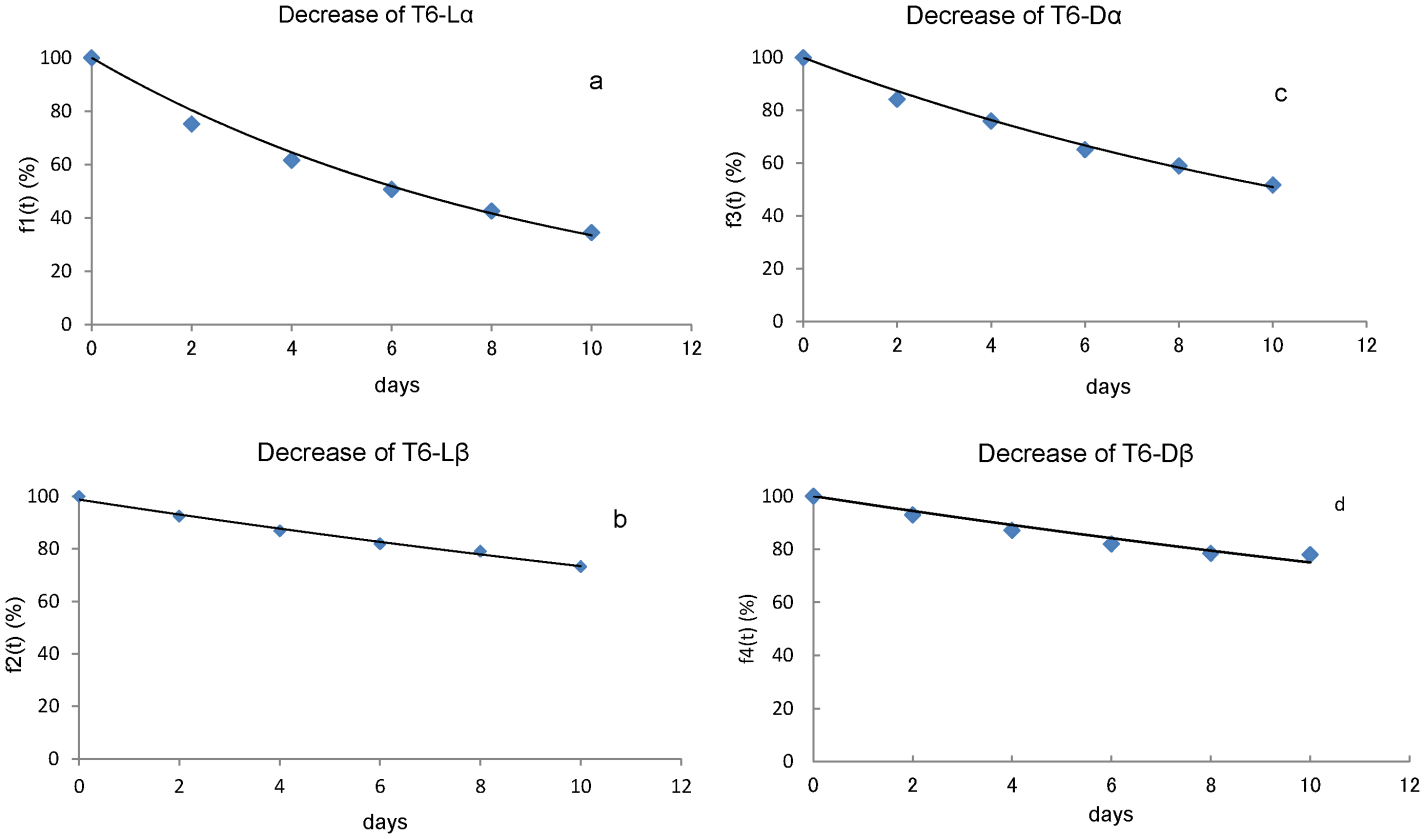
Decrease of 4 Asp isomers at 70°C. Figure 7a: decrease of L-α-Asp with incubation of T6-Lα peptide at 70°C. Figure 7b: decrease of L-β-Asp with incubation of T6-Lβ peptide at 70°C. Figure 7c: decrease of D-α-Asp with incubation of T6-Dα peptide at 70°C. Figure 7d: decrease of D-β-Asp with incubation of T6-Dβ peptide at 70°C. We calculated the rate constant for dehydration from isomers to succinimide at 70°C (k1, k3, k5 and k7) by using fitted curves in this figure. The rate constants at 50, 60, 80 and 90°C were also determined by the same method.

We subjected T6-Lα, -Lβ, -Dα and -Dβ peptides to heating experiments at 50–90°C for 0.4–31 days. Figure 7 shows the decrease in T6-Lα (Figure 7a), T6-Lβ (Figure 7b), T6-Dα (Figure 7c) and T6-Dβ (Figure 7d) peptides at 70°C, respectively. As shown in Figure 7a, the decrease of T6-Lα peptide was the most rapid of the 4 Asp isomers and the amount of T6-Lα peptide reduced to 30% after 10 days of incubation at 70°C. The T6-Dα peptide is the second most unstable (Figure 7c) with 40% remaining after 10 days incubation. The T6-Lβ and T6-Dβ peptides were stable and more than 80% of the peptides remained after 10 days incubation (Figure 7b and d). The T6-Dβ peptide was more stable than T6-Lβ peptide. The result that the β-Asp containing peptide is more stable than the α-Asp containing peptide, is consistent with a previous report [21].

We calculated k1, k3, k5, k7 at 70°Cfrom the derivatives of the fitted curves. As an example, calculation of k1 using the fitted curve (f1(t)) in Figure 7a is as follows:
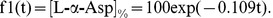
(3 - 1)where the unit of t is day.

Therefore, the derivative of the fitted curve is
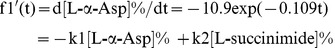
(3 - 2)where the units of k1 and k2 are day^−1^, because the units of t are day in this section.

At t = 0 day, [L-succinimide]_%_ was almost equal to 0%. [L-α-Asp]_%_ was 100% at t = 0 day. Therefore
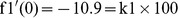
(3 - 3)Therefore, k1 at 70°C was estimated to be 0.109 day^−1^. The rate constants, k3, k5 and k7 at 70°C were determined by the same method.

**Figure 8:**
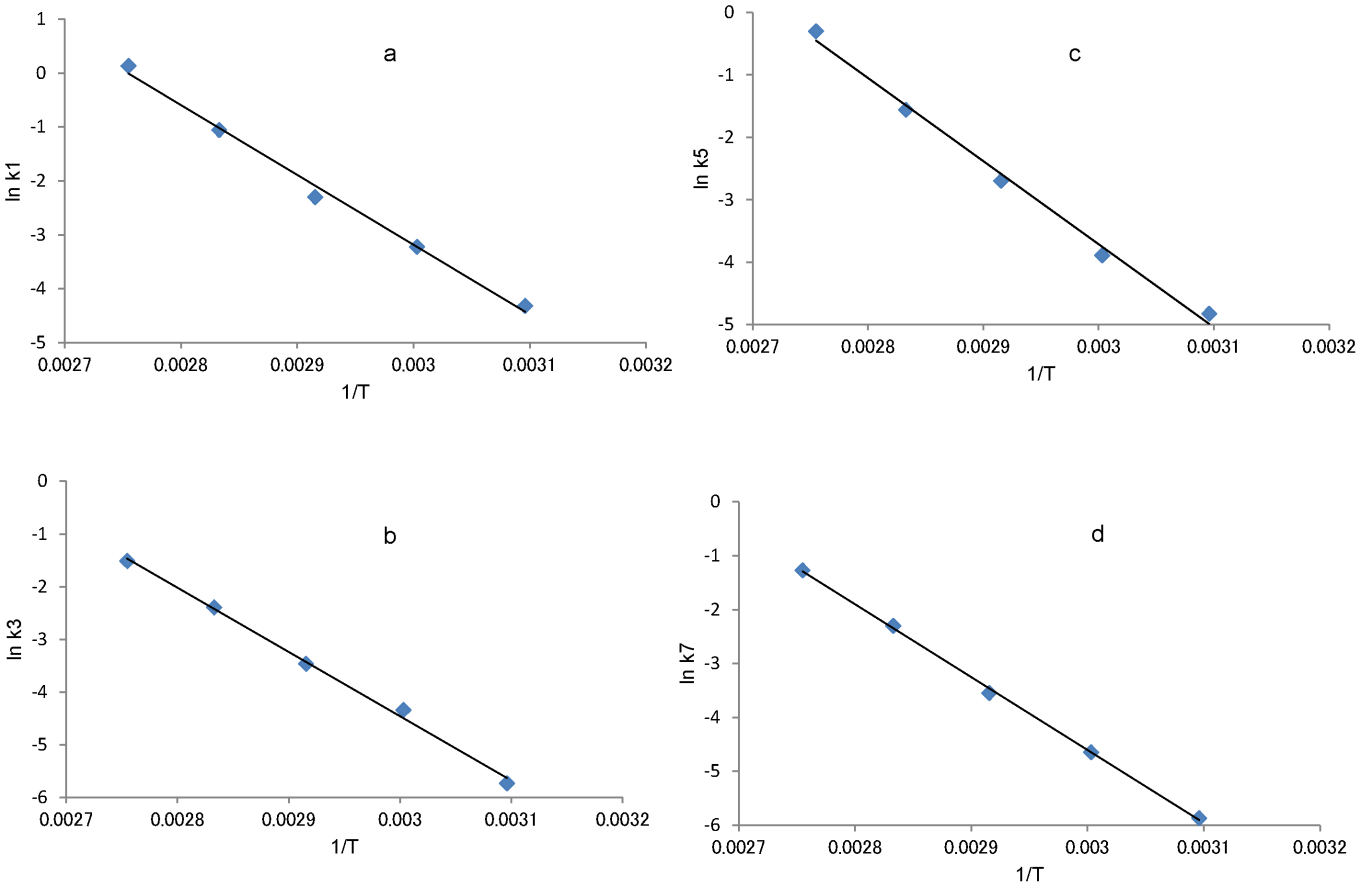
Arrhenius plots of the rate constant for dehydration from isomers to succinimide. Figure 8a Arrhenius plot for k1. Figure 8b shows the Arrhenius plot for k3. Figure 8c shows the Arrhenius plot for k5. Figure 8d shows the Arrhenius plot for k7. Using the fitted line in this figure, we determined rate constants at 37°C.

In addition, the rate constants, k1, k3, k5 and k7 at 50°C–90°C were calculated using the same method. The temperature dependence of these rate constants is shown in an Arrhenius plot (Figure 8). Figures 8a, 8b, 8c and 8d show Arrhenius plots for T6-Lα(k1), T6-Lβ (k3), T6-Dα(k5) and T6-Dβ (k7) peptides respectively. The rate constants k1, k3, k5 and k7 at 37°C were estimated by extrapolation of the Arrhenius plots and are summarized in Table 2. The order of the rate constants is k1>k5>k3>k7 at 37°C. The slopes calculated from the plots were almost the same, indicating that the activation energy for succinimide formation from each of the 4 isomeric peptide is almost equal (about 25 kcal/mol). Therefore, differences inthe rate constants (k1, k3, k5 and k7) may depend on differences in pre-exponential factors.

### Calculation of the Change in Relative Amounts of Asp Isomers in T6 Peptide during a Human Life Span

**Figure 9:**
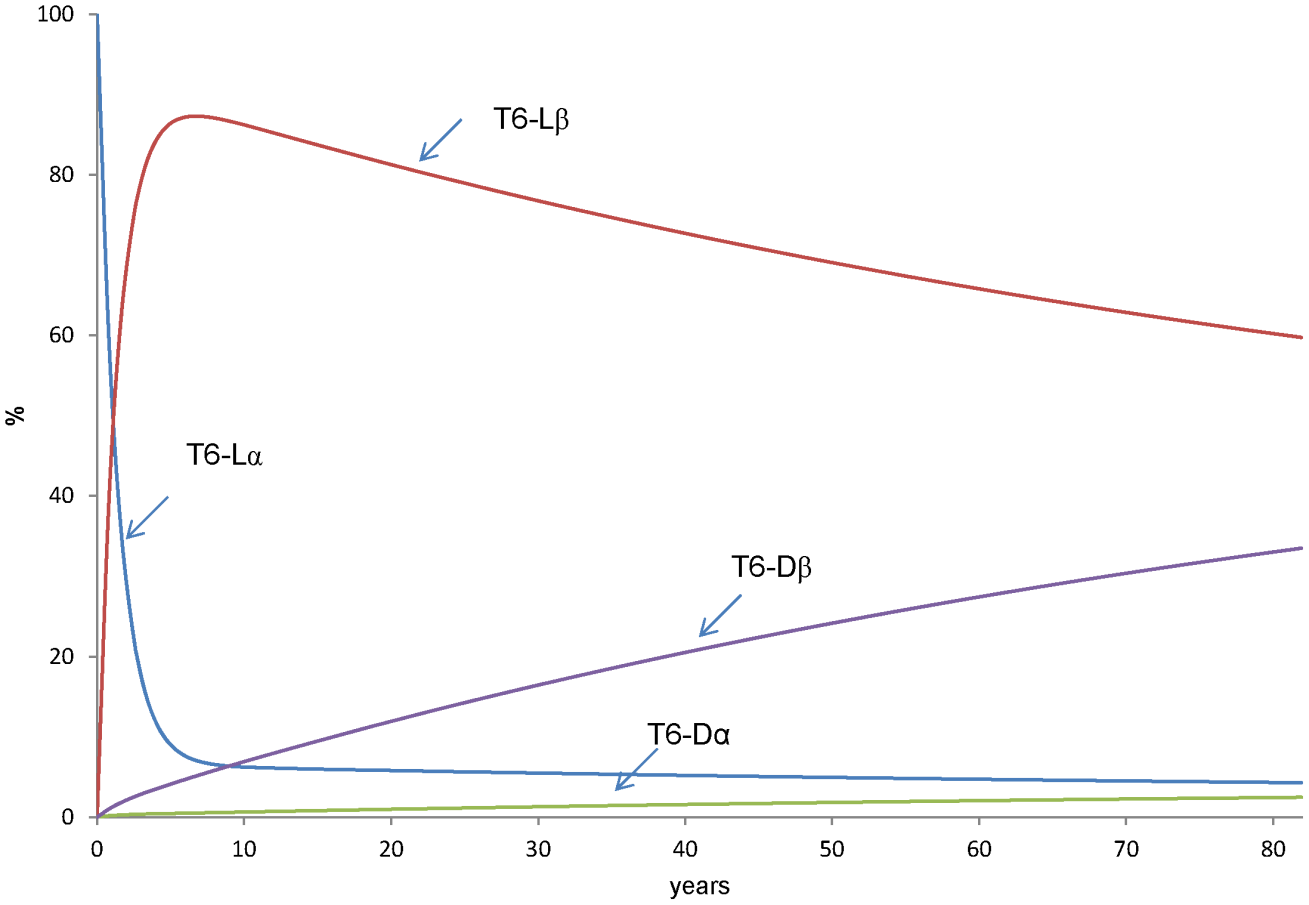
Mathematical simulation of change ofrelative amounts of 4 Asp isomers during human lifespan. Using k1–k10 from Table 2, we calculated the change inrelative amounts of the 4 isomers during a human life span. This calculation was performed using theRunge-Kutta method.

Using the values of k1–k10 (Table 2), we calculated the change in relative amounts of the 4 isomers of Asp 58 in the T6 peptide of αA-crystallin during a human life span. In this calculation, the initial values (t = 0 year) were set as [L-α-Asp]_%_ = 100%, [L-β-Asp]_%_ = 0%, [L-succinimide]_%_ = 0%, [D-α-Asp]_%_ = 0%, [D-β-Asp]_%_ = 0%, [D-succinimide]_%_ = 0%. Figure 9 shows the result of this calculation. At t = 5.5 years, normal L-α-Asp decreases by less than 10%, instead the L-β-Asp dramatically increases by up to 90% and then, L-β-Asp decreases gradually. D-β-Asp increases gradually and the amount of D-β-Asp reaches 32% at 82 years. In contrast, D-α-Asp increases slightly and about 2% of the peptides exist with D-α-Asp at 82 years.

## Discussion

Our previous studies have indicated that specific Asp residues in αA-crystallin (Asp 58 and Asp 151) [2], αB-crystallin (Asp 36 and Asp 62) [3] and βB2-crystallin (Asp 4) [4] in aged human lenses are highly inverted from the L-isomer to the D-isomer and simultaneously the peptide bond between Asp and the next amino acid is isomerized from the normal α-linkage to the β-linkage. The isomerization and inversion of Asp in protein occur through a succinimide intermediate, as shown in Figure 1. However, the rates of isomerization and racemization of the Asp residues in proteins has not been well studied. The purpose of this study was to calculate the rate constants, k1–k10 which were defined in Figure 1, using the T6 peptide corresponding to human αA-crystallin sequence from residues 55 to 65 (T^55^VLD^58^SGISEVR^65^), and to discuss the stability or instability of the Asp isomers in the protein. The T6 peptide and its isomers in which L-α-Asp was replaced with L-β-, D-β- and D-α-Asp at position 58, that is, T6-Lα, T6-Lβ, T6-Dα and T6-Dβ peptides were synthesized. TheT6-Lα, T6-Lβ, T6-Dα and T6-Dβ peptides were clearly separated into 4 peaks by RP-HPLC chromatography as shown in Figure 2a. Using this resolution, we quantified the amounts of the 4 isomeric peptides and estimated the rate constants (k1, k3, k5, k7) for each reaction. It is well known that isomerization and racemization of Asp residues in peptides or proteins proceeds via a succinimide intermediate [22]. In order to calculatek2, k4, k6, k8, k9 and k10, T6-L-suc and T6-D-suc peptides were obtained by heating of T6-Lα and T6-Dα peptides at 90°C for 6 h in water. However, the formation of T6-L-suc and T6-D-suc were not observed in phosphate buffer under the same heating conditions. This result is consistent with a previous study [23] in which it was reported that a succinimidyl peptide decreased from 90% to 0% with increase of pH. Therefore, pH may affect all rate constants (k1–k10) for the conversion of Asp in peptides.

The succinimidyl peptides (T6-L-suc and T6-D-suc) are not stable, therefore, the succinimidyl peptides hydrolyze to α-Asp and β-Asp containing peptides rapidly. As shown in Table 2, the rate constants for the hydrolysis to L-β-Asp from L-succinimide (k4) is about 4.6 times faster than that for the hydrolysis to L-α-Asp from L-succinimide (k2) at 37°C. The rate constants for the hydrolysis to D-β-Asp from D-succinimide (k8) is also 5.5 times higher than that for the hydrolysis to D-α-Asp from D-succinimide (k6) at 37°C. The reason why the succinimide hydrolyzes predominantly to β-Asp rather than to α-Asp is unclear but this result is consistent with many previous reports [22], [24], [25].

The rate constants (k9,k10) for the racemization of the succinimide were calculated by heating of T6-L-suc and T6-D-suc peptides at 37°C (Figure 6). The racemization rate constants k9 and k10 were almost the same, and these constants are fast (Table 2). This may be one of the reasons why the accumulation of D-Asp in proteins in aged human tissues is observed.

In order to determine the rate constants (k1, k3, k5, k7) for succinimide formation in the peptides, we subjected T6-Lα, -Lβ, -Dα and -Dβ peptides to heating experiments at 50–90°C for 10 days. Subsequently, we estimated the rate constants k1, k3, k5 and k7 for succinimide formation in peptides at 37°C using Arrhenius plots.

The rate constants, k1–k10 in Figure 1 were estimated from the heating experiments in Figures 5, 6, 7, 8 and the results are shown in Table 2. Using the estimates of k1–k10, we calculated the amounts of the Asp isomers during a human life span (Figure 9). As shown in Figure 9, normal L-α-Asp decreases to less than 10% within 5.5-years whereas the L-β-Asp dramatically increases up to 90% and then decreases gradually. D-β-Asp increases gradually and the amounts of D-β-Asp reach 32%. at 82 years.

**Figure 10:**
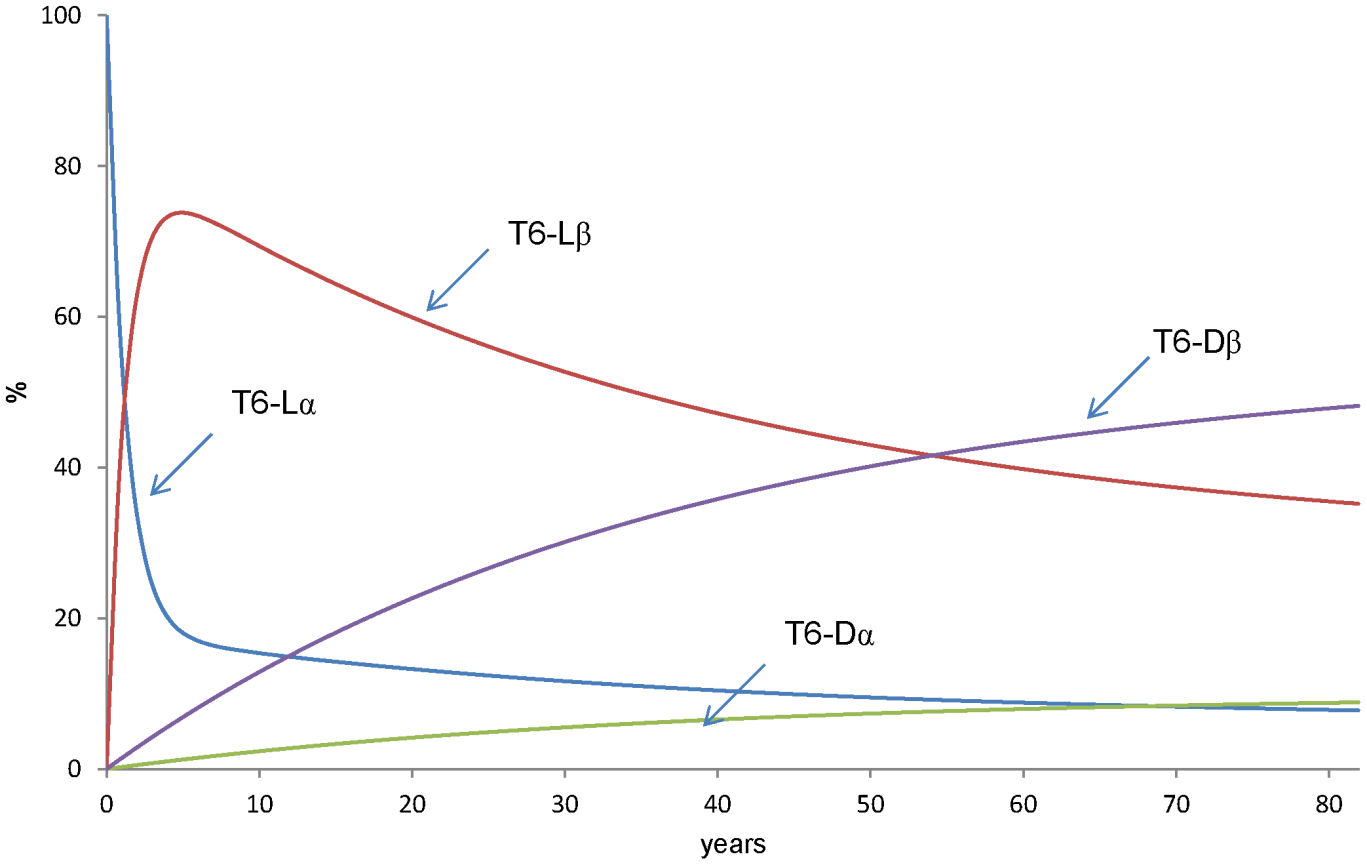
Mathematical simulation assuming k3 = k1 and k7 = k5. We estimated k3 = k1 = 2.22×10^−3^ day^−1^, k7 = k5 = 1.20×10^−3^ day^−1^ in order to compensate for underestimation of the rate constants for dehydration from β-Asp to succinimide (see text).

The lens is a metabolically inactive tissue and therefore significant amounts of abnormal Asp isomers accumulate in lens. Our recent study showed that the ratios of L-α-:L-β: D-β-:D-α-isomers of Asp 58 in αA-crystallin from84-year-old human lens to be about 32%: 23% : 35% :10% by LC-MS analysis [26]. However, the results shown in Figure 9 might not reflect the change in the amounts of the 4 isomers *in vivo*, because we might be underestimating the rate constants for dehydration from β-Asp to succinimide (k3 and k7). Such an underestimation could be due to the rapid rate of hydrolysis from succinimide to β-Asp. Therefore the actual values of k3 and k7 might be near the values of k1 and k5. In fact, Geiger and Clarke [22]estimated k3 = k1 as estimated by decrease of L-α-Asp and k7 = k5 as estimated by decrease of D-α-Asp. We re-estimated the amounts of the isomeric peptides using k3 = k1 = 2.22×10^−3^day^−1^and k7 = k5 = 1.20×10^−3^day^−1^. Figure 10 shows the prediction for the amounts of the 4 Asp isomers at position 58 using the above rate constants during aging. This model may be better than that which gives the results in Figure 9, because the relative amounts of Asp isomers in 80-year-olds calculated from Figure 10 is consistent with the amounts of Asp isomers in αA-crystallin of 84-year-old lenses [26].

The accumulation of abnormal Asp isomers in proteins induces age-related diseases. Our previous study clearly showed thatα-crystallin containing large amounts of abnormal isomers undergoes abnormal aggregation to form massive and heterogeneous aggregates, leading to loss of its chaperone activity [19]. Similarly, we observed the accumulation of abnormal isomers containing proteins in sun-damaged face skin from elderly people [15]. The abnormal protein was localized to the elastic fiber-like structures of dermis from elderly donors with actinic elastosis [20]. These findings indicate that abnormal Asp residues are present widely and arise due to racemization of amino acids in various proteins during the lifespan of the individual. Therefore, it is important to be able to predict the content percentage of 4 isomers as demonstrated in this study.

## Materials and Methods

Synthesis and purification of the T6 peptide and its isomeric peptides containing the four different Asp isomers (T6-Lα, T6L-β, T6-Dα and T6-Dβ peptides).

The T6 peptide corresponding to human αA-crystallin sequence from residues 55 to 65 (T^55^VLD^58^SGISEVR^65^) and its diastereoisomers in which L-α-Asp was replaced with L-β-, D-β- and D-α-Asp at position 58 were synthesized by Fmoc (9-fluorenylmethoxycarbonyl) solid-phase chemistry using an automated solid-phase peptide synthesizer (PSSM-8; Shimadzu, Kyoto, Japan). Fmoc-L-Asp(OtBu)-OH, Fmoc-D-Asp(OtBu)-OH, Fmoc-L-Asp-OtBu and Fmoc-D-Asp-OtBu were used as building blocks to synthesize L-α-, D-α-, L-β- and D-β-isomers, respectively. The coupling reaction was carried out using each Fmoc amino acid (10 equiv), PyBOP (10 equiv), 1-hydroxybenzotriazole (HOBt) (10 equiv) and N-methylmorpholine (7.5 equiv) in dimethylformamide (DMF). The N-terminal Fmoc group was de-blocked with 20% piperidine in DMF. Simultaneous cleavage of the peptide from the resin and removal of the protective groups was achieved by treatment with a cocktail containing 90% TFA, 5% 1,2-ethanedithiol and 5% thioanisole.

The peptides synthesized are as follows;

1. T6-Lα: Thr^55−^Val-Leu- L-α-Asp^58^-Ser-Gly-Ile-Ser-Glu-Val-Arg^65^
2. T6-Lβ: Thr^55−^Val-Leu- L-β-Asp^58^-Ser-Gly-Ile-Ser-Glu-Val-Arg^65^
3. T6-Dα: Thr^55−^Val-Leu- D-α-Asp^58^-Ser-Gly-Ile-Ser-Glu-Val-Arg^65^
4. T6-Dβ: Thr^55−^Val-Leu- D-β-Asp^58^-Ser-Gly-Ile-Ser-Glu-Val-Arg^65^

Crude T6 and the isomeric peptides were purified by RP-HPLC using a C18 column (Capcellpak C18 ACR, 30×250 mm; Shiseido) with a linear gradient of 0–50% acetonitrile in the presence of 0.1% TFA, at a flow rate of 3.0 mL/min, with monitoring at 215 nm. The purity of each peptide was confirmed to be >95% by analytical RP-HPLC and TOF-MS analysis.

### Preparation and Purification of theT6 Peptide Containing Succinimides (T6-L-suc and T6-D-suc)

In order to prepare the T6 peptide containing L- or D-succinimide in which L-α-Asp or D-α-Asp were replaced with succinimide at position 58, the T6-Lα- orT6-Dα-peptides were incubated at 90°C for 6 h in water. T6 peptides containing L- and D-succinimides were obtained from the heating.

1. T6-L-suc: Thr^55^−Val-Leu- L-suc^58^-Ser-Gly-Ile-Ser-Glu-Val-Arg^65^
2. T6-D-suc: Thr^55−^Val-Leu-D-suc^58^-Ser-Gly-Ile-Ser-Glu-Val-Arg^65^

The T6-L-suc and T6-D-suc peptides were purified by RP-HPLC using a C18 column (Capcellpak C18 ACR, 30×250 mm; Shiseido) with a linear gradient of 15–35% acetonitrile in the presence of 0.1% TFA, at a flow rate of 3.0 mL/min, with monitoring at 215 nm. The purity of each peptide was confirmed to be >90% by analytical RP-HPLC and TOF-MS analysis.

#### Matrix-assisted laser desorption/ionization time-of-flight mass spectrometry (MALDI-TOFMS)

All spectra were obtained using a matrix-assisted laser desorption/ionization time-of-flight mass spectrometer (MALDI-TOFMS) (AXIMA-TOF^2^; Shimadzu, Kyoto, Japan). The MALDI-TOFMS spectrometer operated with a 337 nm nitrogen laser and an ion acceleration voltage of 20 kV. Data were collected in positive ion reflection mode. 1 µL of 20 mg/mL α-cyano-4-hydroxycinnamic acid (CHCA) in acetone was applied onto a stainless steel MALDI plate and allow to air dry before addition of 1 µL of the peptide solution.

### Heating Experiments with the T6-Lα, T6-Lβ, T6-Dα and T6-Dβ Peptides

To determine the rate constant for the conversion the Asp isomers to succinimides in the T6 peptide, the T6-Lα, T6-Lβ, T6-Dα and T6-Dβ peptides were dissolved in PBS (pH7.4) and were incubated at 50, 60, 70, 80, and 90°C for 0.4–31 days.

### The Conversion Rate of Asp in T6-Lα, T6-Lβ, T6-Dα and T6-Dβ Peptides

After the incubation of the 4 isomeric peptides, these peptides were subjected to HPLC. Since the four isomeric T6 peptides are clearly separated by RP-HPLC chromatography, we can measure the relative amounts of the peptides produced by the incubation from the peak areas of the 4 isomeric peptides in the chromatogram. The results are as follows:
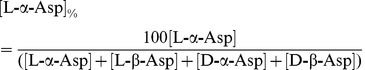
(4 - 1)

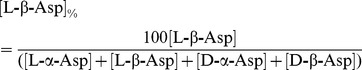
(4 - 2)

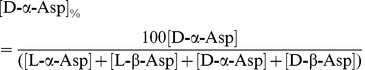
(4 - 3)

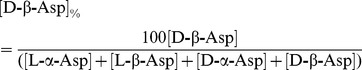
(4 - 4)where [L-α-Asp]_%_, [L-β-Asp]_%_, [D-α-Asp]_%_, and [D-β-Asp]_%_ are relative amounts (%) of L-α-Asp, L-β-Asp, D-α-Asp and D-β-Asp, and [L-α-Asp], [L-β-Asp], [D-α-Asp] and [D-β-Asp] represent the concentration of L-α-Asp, L-β-Asp, D-α-Asp and D-β-Asp respectively, which are calculated from the peak area in the RP-HPLC chromatogram. We plotted the relative amounts against incubation time. Subsequently, we drew fitted curves for this plot as follows:f1(t) for L-α-Asp, f2(t) for L-β-Asp, f3(t) for D-α-Asp, f4(t) for D-β-Asp.

### Kinetic Measurement of Conversion of T6-Lα, T6-Lβ, T6-Dα and T6-Dβ Peptides

The Asp residues invert to isomerize through succinimide intermediates in peptide as shown in Figure 1. The rate constants of the conversion of the peptide isomers can be expressed as follows:
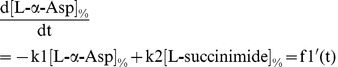
(5 - 1)

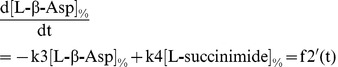
(5 - 2)

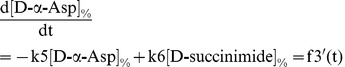
(5 - 3)

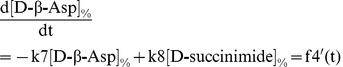
(5 - 4)where [L-succinimide]_%_ and [D-succinimide]_%_ represent the relative amounts of L-succinimide and D-succinimide as a percentage, t is time (day), k1 is the rate constant for the conversion of L-α-Asp to L-succinimide (day^−1^), k2 is the rate constant for the conversionof L-succinimide to L-α-Asp (day^−1^), k3 is the rate constant for the conversion of L-β-Asp to L-succinimide (day^−1^), k4 is the rate constant for the conversion of L-succinimide to L-β-Asp (day^−1^), k5 is the rate constant for the conversion of D-α-Asp to D-succinimide (day^−1^), k6 is the rate constant for the conversion of D-succinimide to D-α-Asp (day^−1^), k7 is the rate constant for the conversion of D-β-Asp to D-succinimide (day^−1^), k8 is the rate constant for the conversion of D-succinimide to D-β-Asp (day^−1^), and f1’(t), f2’(t), f3’(t) and f4’(t) are derivatives of f1(t), f2(t), f3(t) and f4(t). At t = 0 day, [L-α-Asp]_%_ and [L-succinimide]_%_ are 100% and 0% respectively, therefore, Eq. (5-1) can be expressed as:



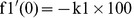
(6 - 1)Similarly, Eq. (5–2), (5–3) and (5–4) can be expressed as follows at t = 0:
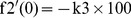
(6 - 2)

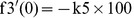
(6 - 3)

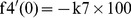
(6 - 4)


We determined k1, k3, k5 and k7 at five temperatures (50, 60, 70, 80 and 90°C) by using Eq. (6-1), (6-2), (6-3), (6-4). The Arrhenius equation shown below gives the activation energy of the degradation reaction.
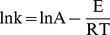
(7)where k is rate constant, E is the activation energy of this reaction (kcal/mol), R is the gas constant (1.99×10^−3^ kcal/Kmol), A is the frequency constant (day^−1^), and T is the absolute temperature (K). From Eq. (7), we calculated k1, k3, k5 and k7 at 37°C using k1, k3, k5 and k7 from five temperatures (50, 60, 70, 80 and 90°C).

### Heating Experiment with T6-L-suc and T6-D-sucpeptides

To determine the rate constant for the conversion from the succinimide intermediate to the peptides containing Asp isomers and the racemization rate of succinimide, samples were dissolved in PBS (pH7.4) and incubated at 37°C.

### Measurement of the Production of Isomeric Peptides from the T6-L-suc and T6-D-suc Peptides

After incubation, the T6-L-suc and T6-D-sucpeptideswere subjected to RP-HPLC. We measured the amounts of theT6-Lα, T6-Lβ, T6-Dα and T6-Dβ, T6-L-suc and T6-D-suc peptides from the peaks areas in the RP-HPLC chromatogram. The results are as follows:
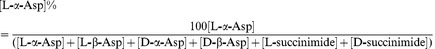
(8 - 1)

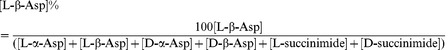
(8 - 2)

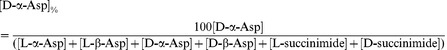
(8 - 3)

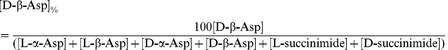
(8 - 4)

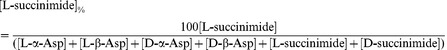
(8 - 5)

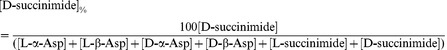
(8 - 6)where [L-succinimide] and [D-succinimide] represent concentration (peak area in chromatogram) of L-succinimide and D-succinimide. We plotted percentage content of the 4 isomers against incubation time. Subsequently, we drew fitted curves of these plots as follows: F1(t) for increase of L-α-Asp from L-succinimide, F2(t) for increase of L-β-Asp from L-succinimide, F3(t) for increase of D-α-Asp from D-succinimide, F4(t) for increase of D-β-Asp from D-succinimide. G1(t) for increase of D-Asp from L-succinimide, G2(t) for increase of L-Asp from D-succinimide.

### Kinetic Measurement of Increase of T6-Lα, T6-Lβ, T6-Dα and T6-Dβ

The change of percentage content of isomers can be expressed as follows:
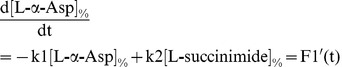
(9 - 1)

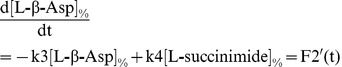
(9 - 2)

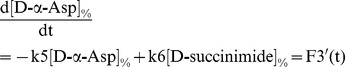
(9 - 3)

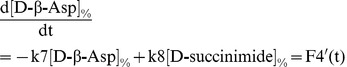
(9 - 4)where the units of k1–k8 are min^−1^ in this section, F1’(t), F2’(t), F3’(t) and F4’(t) are derivatives of F1(t), F2(t), F3(t) and F4(t). Because the dehydration rate of isomers is 1000–10000 times slower than the hydrolysis rate of succinimide, Eq. (9–1), (9–2), (9–3) and (9–4) can be expressed as follows at t = 0:
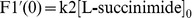
(10 - 1)

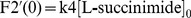
(10 - 2)

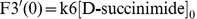
(10 - 3)

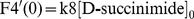
(10 - 4)where [L-succinimide]_0_ and [D-succinimide]_0_ are relative amounts (%) of L- succinimide and D-succinimide at t = 0 min. We determined k2, k4, k6 and k8 at 37°C by using Eq. (10–1), (10–2), (10–3) and (10–4). Then the units of k2, k4, k6 and k8 (min^−1^) was converted to day^−1^.

### Kinetic Measurement of Racemization of Succinimide

Racemization of succinimides in T6 peptide can expressed as follows;
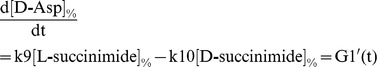
(11 - 1)

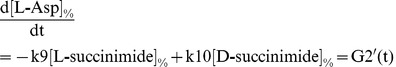
(11 - 2)where [D-Asp]_%_ is relative amounts of D-Asp (%), [L-Asp]_%_ is relative amounts of L-Asp (%), k9 is rate constant for L-succinimide to D-succinimide (min^−1^), k10 is rate constant for conversion of D-succinimide to L-succinimide (min^−1^), and G1’(t) and G2’(t) are derivative of G1(t) and G2(t) respectively. Because [D-succinimide]_%_is probably 0 at t = 0, Eq. (11-1) can be expressed as follows at t = 0 min :
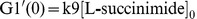
(12 - 1)Eq. (11-2) can be expressed as follows at t = 0:
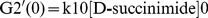
(12 - 2)We determined k9 and k10 by using Eq. (12–1) and (12–2). Then, the units of k9 and k10 (min−1) were converted to day−1.

### Calculation of the Relative Amounts of the Asp Isomers in T6 Peptide under Physiological Conditions

Using these rate constants, we calculated the change in relative amounts of the Asp isomers in T6 peptide during a human life span. This calculation was performed using the Runge-Kutta method (see Appendix S1).

## Supporting Information

Appendix S1 (DOCX)
