# Supplementary material for: Kinetics of Isomerization and Inversion of Aspartate 58 of αA-Crystallin Peptide Mimics under Physiological Conditions
Source: PLoS One. 2013 Mar 7;8(3):e58515. doi: 10.1371/journal.pone.0058515 (PMC3591338; doi:10.1371/journal.pone.0058515)
Supplement: Appendix S1 — (DOCX) [file pone.0058515.s001.docx]

**Appendix**

Using rate constants (day^-1^) in table 2, we calculated the change in relative amounts of the Asp isomers in T6 peptide during a human life span. The change in relative amounts of the Asp isomers is described as solutions of simultaneous ordinary differential equations as follows:

$$\begin{matrix} \frac{\text{d[La]}}{\text{dt}}\text{ =-k1[La]+k2[Ls] (1-1)} \\ \frac{\text{d[Lb]}}{\text{dt}}\text{ =-k3[Lb]+k4[Ls] (1-2)} \\ \frac{\text{d[Ls]}}{\text{dt}}\text{ = -(k2+k4+k9)[Ls]+k1[La]+k2[Lb]+k10[Ds] (1-3)} \\ \frac{\text{d[Da]}}{\text{dt}}\text{ = -k5[Da]+k6[Ds] (1-4)} \\ \frac{\text{d[Db]}}{\text{dt}}\text{ = -k7[Db]+k8[Ds] (1-5)} \\ \frac{\text{d[Ds]}}{\text{dt}}\text{ =-(k6+k8+k10)[Ds]+k5[Da]+k7[Db]+k9[Ls] (1-6)} \end{matrix}$$

where [La], [Lb], [Ls], [Da], [Db] and [Ds] are calculated relative amounts (%) of L-α-Asp, L-β-Asp, L-succinimide, D-α-Asp, D-β-Asp and D-succinimide and t is time (day). However, these simultaneous ordinary differential equations are too difficult to solve. Therefore this calculation was performed using the Runge-Kutta method as follows:

1. *Definition of functions based on simultaneous ordinary differential equations.*

We defined the function to calculate the slope during a short time

based on Eq. (1-1) - Eq. (1-6) as follows:

$$\begin{matrix} \text{-k1[La]+k2[Ls]=f}_{\text{La}}\text{([La], [Ls]) (2-1)} \\ \text{-k3[Lb]+k4[Ls]=f}_{\text{Lb}}\text{([Lb],[Ls]) (2-2)} \\ \text{-(k2+k4+k9)[Ls]+k1[La]+k2[Lb]+k10[Ds]=f}_{\text{Ls}}\text{([La],[Lb],[Ls],[Ds]) (2-3)} \\ \text{-k5[Da]+k6[Ds]=f}_{\text{Da}}\text{([Da],[Ds]) (2-4)} \\ \text{-k7[Db]+k8[Ds]=f}_{\text{Db}}\text{([Db],[Ds]) (2-5)} \\ \text{-(k6+k8+k10)[Ds]+k5[Da]+k7[Db]+k9[Ls]=f}_{\text{Ds}}\text{([Ls], [Da], [Db], [Ds]) (2-6)} \end{matrix}$$

1. *Calculation of 4 sets of slopes from n step to n+1 step*

The slopes during the short time were calculated as follows:

where [La]_n_, [Lb]_n_, [Ls]_n_, [Da]_n_, [Db]_n_ and [Ds]_n_ are calculated relative amounts (%) of L-α-Asp, L-β-Asp, L-succinimide, D-α-Asp, D-β-Asp and D-succinimide at n step, n is steps, h is increment size of time in 1 step.

1. *Calculation of Mean slopes from n step to n+1 step*

The average value of slopes in the above (s_La1_ - s_Ds4_) was calculated as follows:

1. *Calculation of relative amounts of isomers for n+1 step using mean slopes*

The time at n+1 step is

Using mean slopes (S_La_-S_Ds_), the relative amounts of T6 isomers at n+1 step can be described as follows:

$$\begin{matrix} \text{[La]}_{\text{n+1}}\text{=[La]}_{\text{n}}\text{+S}_{\text{La}}\text{h (8-2)} \\ \text{[Lb]}_{\text{n+1}}\text{=[Lb]}_{\text{n}}\text{+S}_{\text{Lb}}\text{h (8-3)} \\ \text{[Ls]}_{\text{n+1}}\text{=[Ls]}_{\text{n}}\text{+S}_{\text{Ls}}\text{h (8-4)} \\ \text{[Da]}_{\text{n+1}}\text{=[Da]}_{\text{n}}\text{+S}_{\text{Da}}\text{h (8-5)} \\ \text{[Db]}_{\text{n+1}}\text{=[Db]}_{\text{n}}\text{+S}_{\text{Db}}\text{h (8-6)} \\ \text{[Ds]}_{\text{n+1}}\text{=[Ds]}_{\text{n}}\text{+S}_{\text{Ds}}\text{h (8-7)} \end{matrix}$$

Where [La]_n+1_, [Lb]_n+1_, [Ls]_n+1_, [Da]_n+1_, [Db]_n+1_ and [Ds]_n+1_ are calculated relative amounts of L-α-Asp, L-β-Asp, L-succinimide, D-α-Asp, D-β-Asp and D-succinimide at n+1 step( t= (n+1)h).

Using Eq. (2-1) –Eq.(8-7) repetitively, we calculated relative amounts of isomers during a human life span. Then the unit of t (day) was converted to years, in order to display in a clearer manner.
